# Relationship between expansion of the myocardial interstitial space and ventricular performance in patients with pulmonary hypertension

**DOI:** 10.1186/1532-429X-18-S1-P291

**Published:** 2016-01-27

**Authors:** Elena Pena, Rebecca E Thornhill, Lin Yassin Kassab, Carole Dennie, Girish Dwivedi, Alexander Dick, Lisa Mielniczuk

**Affiliations:** 1 grid.412687.e0000000096065108Medical Imaging, The Ottawa Hospital, Ottawa, ON Canada; 2 grid.28046.380000000121822255Radiology, University of Ottawa, Ottawa, ON Canada; 3 grid.34428.39000000041936893XCarleton University, Ottawa, ON Canada; 4 grid.28046.380000000121822255Division of Cardiology, Department of Medicine, University of Ottawa Heart Institute, Ottawa, ON Canada

## Background

Diffuse interstitial fibrosis is a common end point in heart failure from multiple etiologies. The post contrast T1 time, extracellular volume (ECV) and partition coefficient (λ) of gadolinium-based contrast media have been used to estimate expansion of myocardial interstitial space, as a surrogate of interstitial fibrosis. The aim of the study was to explore the relationship between T1-based estimates of interstitial expansion and both right (RV) and left (LV) ventricular functional parameters in pulmonary hypertension (PH).

## Methods

We prospectively recruited 8 patients with a diagnosis of PH (pulmonary arterial hypertension, n=5; chronic thromboembolic pulmonary hypertension, n=3) who underwent CMR at 3T. CMR studies included functional (bSSFP) cine imaging for assessment of ventricular function. Phase contrast velocity encoded cine imaging was obtained perpendicular to the main pulmonary artery for RV stroke volume assessment. T1 maps (saturation recovery single-shot acquisition (SASHA) were acquired both pre- and 15 minutes after administration of 0.2mmol/kg of Gadobutrol. For each patient, a blood sample was acquired prior to scanning to evaluate hematocrit. Whole-LV as well as septal values of post-contrast T1, ECV and λ were evaluated offline. The relationships between each T1-based estimate and functional parameters were investigated using Spearman's rank correlation (rho).

## Results

Significant positive correlations were found between septal post contrast T1 time and RV stroke volume indexed (RVSVI) (rho=0.86,p=0.007) and between λ and RV end-diastolic volume indexed (RVEDVI) (rho=0.74,p=0.04) as well as between whole-LV post contrast T1 times and LV ejection fraction (LVEF) (rho=0.83,p=0.04). There were no significant correlations between ECV values and any functional parameters (p>0.05 for each).

Trends towards positive correlations between whole-LV post-contrast T1 and LV stroke volume indexed (rho=0.69,p=0.06), RVSVI (rho=0.62, p = 0.10) and RV ejection fraction (rho=0.60,p=0.12), and towards negative correlations with RV end systolic volume indexed (rho=-0.60,p=0.12) were found. There was also a trend towards positive correlation between septal post contrast T1 and LVEF (rho=0.60, p = 0.12). Furthermore, trends toward negative correlations between septal λ and both RV stroke volume (rho=-0.62, p = 0.10) and RV cardiac output (rho=-0.60,p=0.12) were revealed.

## Conclusions

λ increases with dilatation of the RV and shorter post contrast T1 times are seen in patients with increased RV end diastolic volumes and decreased stroke volumes, as well as lower LVEF reflecting a link between diffuse interstitial fibrosis and biventricular functional impairment. Post contrast T1 times and λ may be non-invasive biomarkers of increased interstitial fibrosis seen in maladaptive stages of the disease when progressive dilatation of the RV results in RV systolic dysfunction leading to impairment of LV filling.

**Figure 1:**
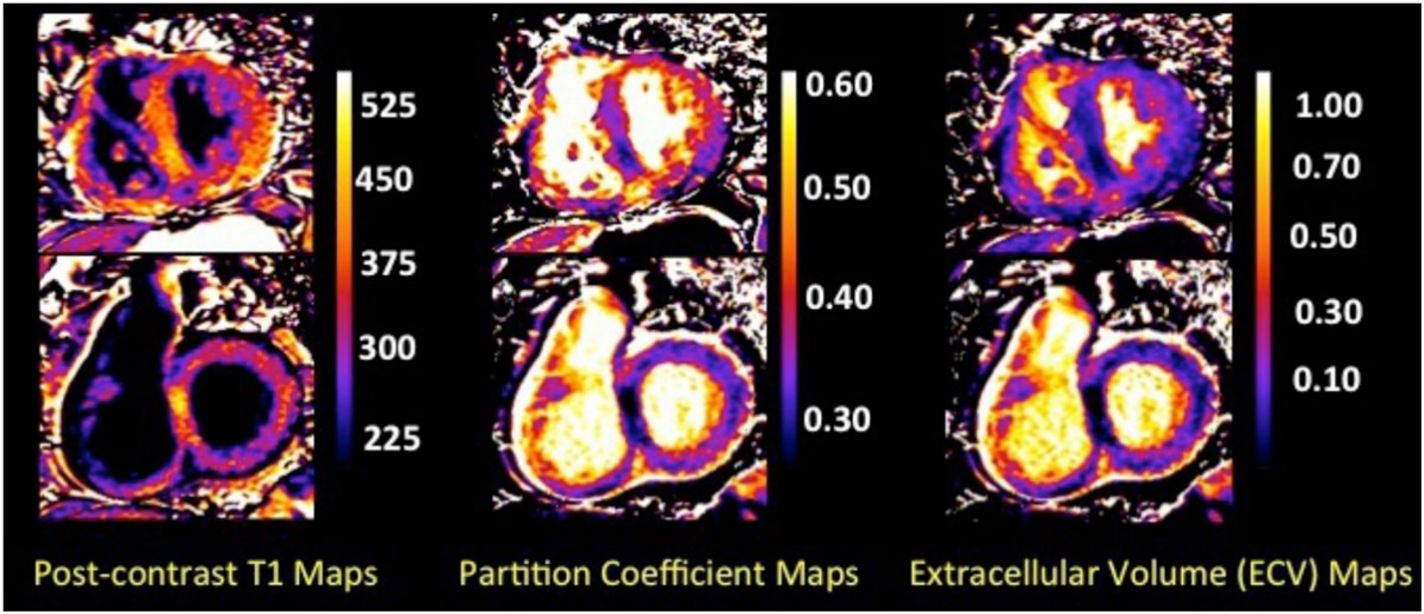
**Post contrast T1, partition coefficient and extracellular volume maps in a patient with pulmonary hypertension**.

